# Multiphoton microscopy imaging of fibrous meningiomas based on the combination of multichannel mode and lambda mode

**DOI:** 10.3389/fnins.2025.1680408

**Published:** 2025-10-31

**Authors:** Linghan You, Linjing Shi, Yuqing Huang, Lingxin Pan, Shiying Zheng, Yingyuan Wang, Zanyi Wu, Xingfu Wang, Jianxin Chen, Na Fang

**Affiliations:** ^1^School of Medical Technology and Engineering, Fujian Medical University, Fuzhou, China; ^2^Department of Neurosurgery, The First Affiliated Hospital of Fujian Medical University, Fuzhou, China; ^3^Department of Pathology, The First Affiliated Hospital of Fujian Medical University, Fuzhou, China; ^4^Key Laboratory of OptoElectronic Science and Technology for Medicine of Ministry of Education, Fujian Provincial Key Laboratory of Photonics Technology, Fujian Normal University, Fuzhou, China

**Keywords:** multiphoton microscopy, fibrous meningiomas, two-photon excitation fluorescence, second harmonic generation, spectral analysis

## Abstract

Fibrous meningiomas, known for their dense and tough texture, present unique challenges in diagnosis and surgical treatment. This study explores the potential of multiphoton microscopy (MPM) for visualizing the microstructures of fibrous meningiomas by combining multichannel and lambda modes. Using MPM, we imaged 14 fibrous meningioma samples collected from neurosurgical procedures. The multichannel mode captured second harmonic generation (SHG) and two-photon excitation fluorescence (TPEF) signals, while the lambda mode provided detailed spectral imaging across 32 channels. Image analysis algorithms were developed to quantify collagen content and assess morphological features. Spectroscopic analysis revealed the intrinsic components of fibrous meningiomas, with collagen being the most abundant component (relative ratio: 0.952), followed by structural proteins (0.502), free-form NADH (0.393), FAD (0.199), lipopigments (0.198), protein-bound NADH (0.105), porphyrin derivatives I (0.104), and porphyrin derivatives II (0.015). The combined spectral images also provided high-contrast and high-resolution views of the tumor microenvironment. Quantitative analysis showed that the average collagen content in fibrous meningioma tissues was 0.537 ± 0.131 using SHG imaging and 0.503 ± 0.133 using combined 32-channel spectral imaging. With the advancement of fiber optic technology and multiphoton endoscopy, multiphoton microscopy holds promise as a new technology for clinically diagnosing fibrous meningiomas.

## 1 Introduction

Meningiomas are the second most common intracranial neoplasm after gliomas, representing approximately 30% of primary central nervous system tumours (Wang et al., 2024; Simon and Gousias, 2024). The 2021 World Health Organization classification recognizes 15 histological subtypes, among which fibrous meningioma is one of the most frequently encountered (Louis et al., 2021; Soni et al., 2025). Gross-total resection remains the cornerstone of therapy; however, the dense, collagen-rich matrix that defines fibrous meningiomas confers a firm, rubbery consistency. This distinctive texture complicates surgical manipulation, increases operative time and elevates the risk of incomplete resection, particularly for large lesions. Consequently, accurate pre- or intra-operative identification of fibrous meningioma is essential for tailoring the surgical strategy and for providing reliable prognostic information (Sughrue et al., 2010; Erkmen et al., 2005).

Advances in computed tomography (CT), positron-emission tomography (PET), single-photon emission computed tomography (SPECT) and magnetic resonance imaging (MRI) have greatly facilitated the detection and macroscopic characterisation of meningiomas (Villanueva-Meyer et al., 2017). Yet the spatial resolution of these modalities remains insufficient to resolve micron-scale tissue architecture. Histopathology is regarded as the gold standard for definitive diagnosis; however, conventional formalin fixation, paraffin embedding and staining are labor-intensive and require several hours to complete (Supplementary Figure 1A), precluding real-time intraoperative guidance. To expedite diagnostic workflows, a number of label-free imaging techniques—including photoacoustic imaging and optical coherence tomography—have been explored (Monaco and Friedlander, 2012; Yu et al., 2019). However, neither technique produces images with sufficient resolution and contrast to adequately demonstrate the microstructures of meningioma tissue.

Multiphoton microscopy (MPM) which is based on two-photon excited fluorescence (TPEF) and second harmonic generation (SHG) has garnered significant attention, particularly in tumor diagnosis and treatment, due to its high resolution and suitability for real-time *in situ* detection of microstructural changes in human tissues (Cicchi et al., 2010; Chen et al., 2011; Paoli et al., 2009; Kirkpatrick et al., 2007; Tewari et al., 2011; Wang et al., 2009; Chen et al., 2014; Zhuo et al., 2012; König, 2008). TPEF is an absorptive process in which the fluorophore absorbs two photons with lower energy simultaneously and emits a single photon of fluorescence with higher energy. SHG is a coherent scattering process in which two photons with lower energy are combined to create a single photon of exactly twice the lower energy. For meningiomas, intracellular nicotinamide adenine dinucleotide (NADH) and flavin adenine dinucleotide (FAD) emit two-photon excitation fluorescence, while fibrillar collagen produces second-harmonic generation signals without exogenous labels (Kasischke et al., 2004; Chia et al., 2008; Zipfel et al., 2003). This intrinsic contrast makes MPM especially suitable for visualising the collagen-rich matrix of fibrous meningiomas. In addition, established image-analysis routines originally developed for neuron detection, collagen fibre mapping and metabolic assessment can be directly applied to MPM data, yielding both morphological and quantitative information on meningioma microstructure (Wang et al., 2020; Kistenev et al., 2019; Liu et al., 2018).

To date, MPM studies on meningiomas have concentrated on discriminating them from other intracranial tumours or on histological grading (Zanello et al., 2017; Zanello et al., 2017); fibrous meningioma has not been examined specifically. Here we fill that gap by integrating spectral analysis, TPEF, SHG and automated image analysis to characterize this collagen-rich subtype (Supplementary Figure 1B). We first identify endogenous molecular components by lambda-mode of MPM, next visualize tissue architecture with multichannel TPEF/SHG imaging, and finally quantify collagen content and microstructural features with a custom MATLAB algorithm.

## 2 Materials and methods

### 2.1 MPM imaging

The previously described MPM system, depicted in Supplementary Figure 2, was utilized in this study (Zhuo et al., 2006). This system comprises a Zeiss LSM 510 microscope (Jena, Germany) and a Ti:sapphire femtosecond laser (Mira 900-F; Coherent, Inc.) tunable from 700 to 980 nm (Chameleon Ultra, Coherent, Inc., Santa Clara, CA, USA). A 63 × Plan-Apochromat oil immersion objective (NA = 1.4, Zeiss, Jena, Germany) was employed to focus the excitation beam onto the sample and collect the backward two-photon excited fluorescence (TPEF) and second harmonic generation (SHG) signals.

The system has two imaging modes: multichannel mode and lambda mode. Both utilize the same META detector. In multichannel mode, it has eight independent channels which are also using the same META detector. For this study, two channels were selected: one for collecting SHG signals (color-coded green) in the wavelength range of 389–419 nm, and the other for collecting TPEF signals (color-coded red) in the range of 430–716 nm. In lambda mode, the detector can simultaneously capture spectral resolved images and the corresponding emission spectra. The spectral images were obtained across a wavelength range of 382–714 nm, using 32 channels with an 11-nm interval through emission lambda stacks. Data from 32 channels were further analyzed by calculating the mean value of the region of interest (ROI). The excitation wavelength was set at 810 nm, with a power output ranging from 5 to 10 mW for both multichannel mode and lambda mode. Both TPEF/SHG images and combined 32-channel spectral images were acquired at a rate of 2.56 μs per pixel, with a 12-bit pixel depth. The imaging depth under these conditions was approximately 100 μm in fibrous meningioma tissue.

### 2.2 Sample preparation

This study was approved by the Clinical Research Screening Committee for Studies Involving Human Subjects at Fujian Medical University and obtained informed consent from all participants. A total of 14 fibrous meningioma samples were collected from neurosurgical procedures conducted at the First Affiliated Hospital of Fujian Medical University between 2022 and 2024. Fresh specimens, obtained during brain surgery, were immediately placed in a standard pathological transport container, covered with ice, and promptly transported to the pathology laboratory within 30 min. The samples were then embedded in Optimal Cutting Temperature (OCT) compound and stored at −80 °C until sectioning using a Leica CM3050 cryostat microtome.

**Figure 1:**
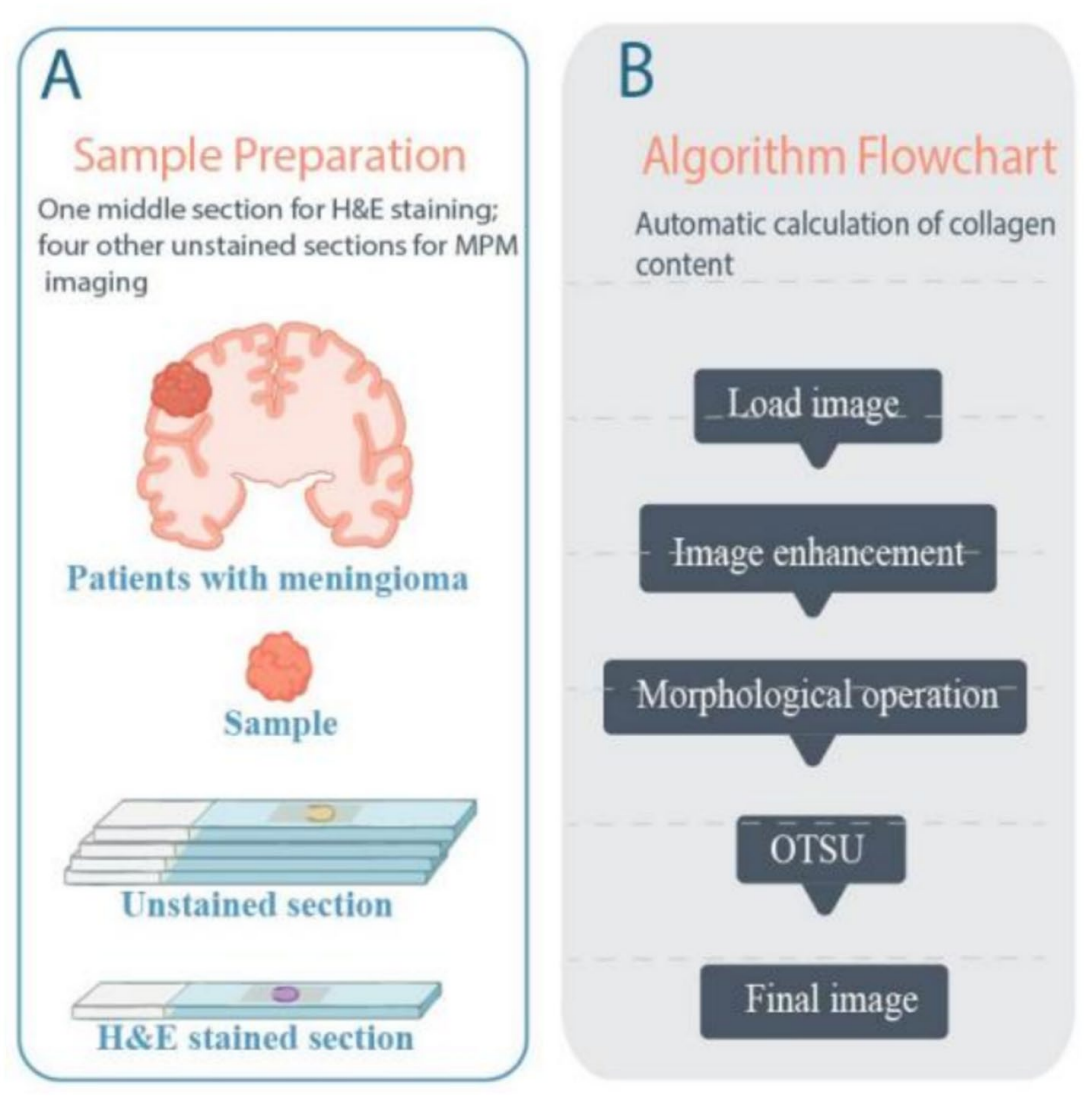
Sketch of sample preparation and flowchart for automatic calculation of collagen content. **(A)** Sketch of sample preparation; **(B)** Flowchart of automatic calculation of collagen content.

As shown in Figure 1A, each specimen was sectioned into five serial cryostat sections. Sections 1, 2, 4, and 5, each approximately 20 μm thick, were used for MPM imaging. Section 3, with a thickness of 4–6 μm, was stained with hematoxylin and eosin (H&E) to obtain histological images and for comparative validation of the MPM imaging results. During MPM imaging, the fresh cryosections were mounted between a cover slip and a microscope slide and perfused with phosphate-buffered saline (PBS) solution to prevent dehydration or shrinkage. The pathological diagnosis of fibrous meningiomas was independently confirmed by two professional neuropathologists, using both H&E images for pathology diagnosis and MPM images for a blind test.

### 2.3 Automatic calculation of collagen content

In our experiment, we developed a custom program using MATLAB R2023b (The MathWorks, Inc.) to automatically quantify collagen content in fibrous meningioma tissues. The program integrates SHG images and combined 32-channel spectral images to achieve accurate segmentation and quantitative analysis of collagen fibres.

To calculate the collagen content, defined as the proportion of collagen pixels relative to the total number of pixels in the image, the SHG and combined 32-channel spectral images were processed through an integrated algorithm. The original images were first enhanced to improve contrast and visibility by adjusting the intensity distribution, thereby highlighting collagen fibers against the background. Subsequently, Otsu’s thresholding method was applied to segment collagen and non-collagen regions by automatically determining the optimal threshold value based on the image histogram. To refine the segmentation, mathematical morphology operations, including opening and closing, were employed to remove excess noise and background artifacts, ensuring clean and well-defined collagen regions. Finally, the collagen content was calculated by counting the number of collagen pixels and dividing by the total number of pixels in the image. The entire process is illustrated in Figure 1B, which serves as a flowchart of the algorithm.

## 3 Results

### 3.1 Multiphoton spectral analysis of fibrous meningiomas

**Figure 2:**
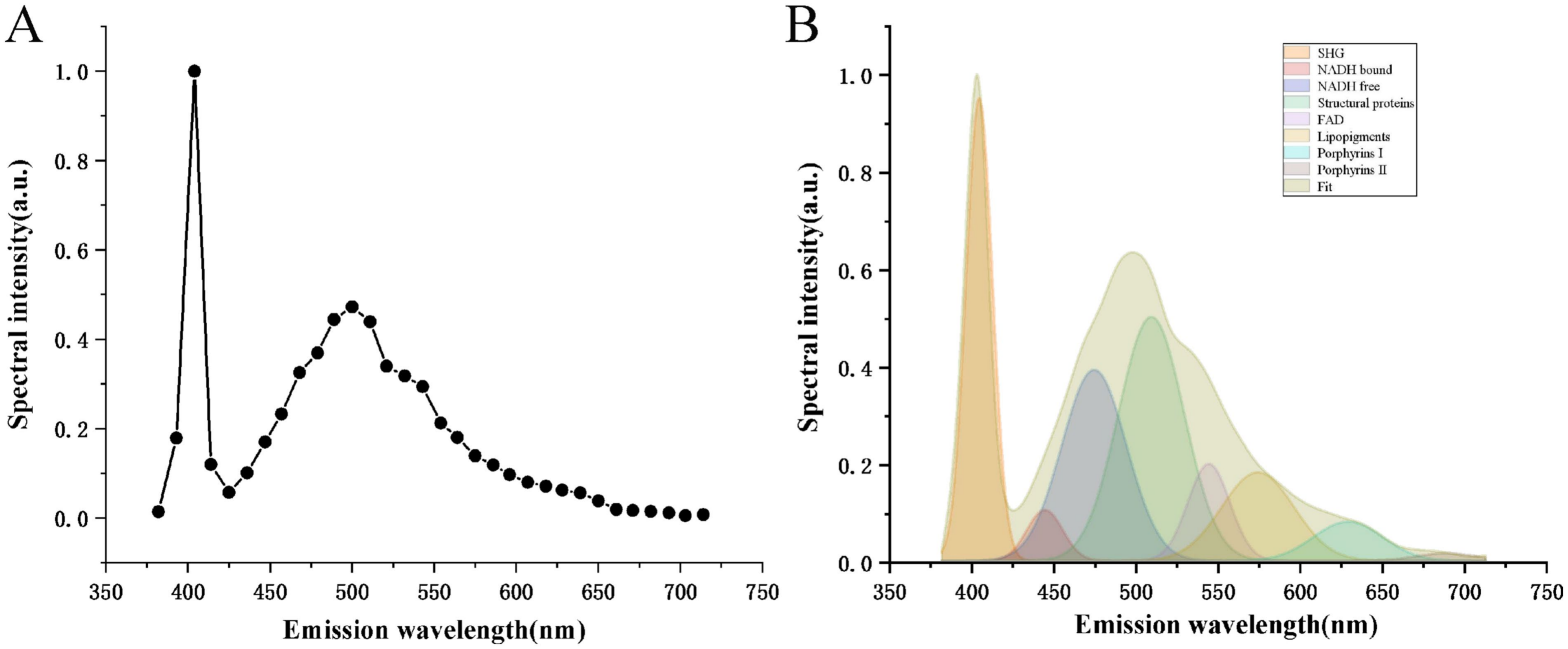
Multi-photon spectral analysis of fibrous meningiomas. **(A)** Normalized multi-photon emission spectra of fibrous meningiomas; **(B)** Multi-peak fitting of fibrous meningiomas.

To evaluate whether multiphoton spectroscopy can resolve the intrinsic composition of fibrous-meningioma microstructure, we acquired lambda-mode MPM spectra from three randomly selected regions of each specimen. All spectra were recorded under identical excitation/collection conditions and normalised with Origin 2018; the averaged result is shown in Figure 2A.

The normalized emission spectra exhibit two prominent peaks at 405 nm and 510 nm. Additionally, seven minor peaks are observed at wavelengths of 425 nm, 475 nm, 545 nm, 575 nm, 630 nm, and 690 nm. Notably, a strong second harmonic generation (SHG) signal is detected at 405 nm, which is exactly half the wavelength of the excitation light. As previously reported, collagen fibers are known to produce SHG signals, indicating that the 405 nm peak originates from collagen in fibrous meningioma tissues. The emission peak at 510 nm, which is the most intense, corresponds to structural proteins. The peaks at 425 nm, 475 nm, 545 nm, and 575 nm are attributed to protein-bound NADH, free-form NADH, FAD, and lipopigments, respectively, within fibrous meningioma cells. The peaks at 630 nm and 690 nm are associated with porphyrins I and porphyrins II.

To further analyze the spectra, we employed a multi-peak fitting program based on the MATLAB language to identify and quantify the different endogenous molecules that emit fluorescence in fibrous meningioma tissues (Ibrahim et al., 2016; Haidar et al., 2015). Figure 2B displays the fitted curves for these endogenous molecules. As shown in the figure, collagen fibers are the most abundant component in the tissue, followed by structural proteins, free-form NADH, FAD, lipopigments, protein-bound NADH, lanthanide derivatives I, and porphyrin derivatives II. Their respective ratios are 0.952: 0.502: 0.393: 0.199: 0.198: 0.105: 0.104: 0.015.

These results demonstrate that the combination of MPM and multi-peak fitting techniques can be effectively used to perform label-free, quantitative, and qualitative analysis of the intrinsic composition of fibrous meningioma tissues.

### 3.2 Visualization of fibrous meningiomas using multichannel-mode of MPM

**Figure 3:**
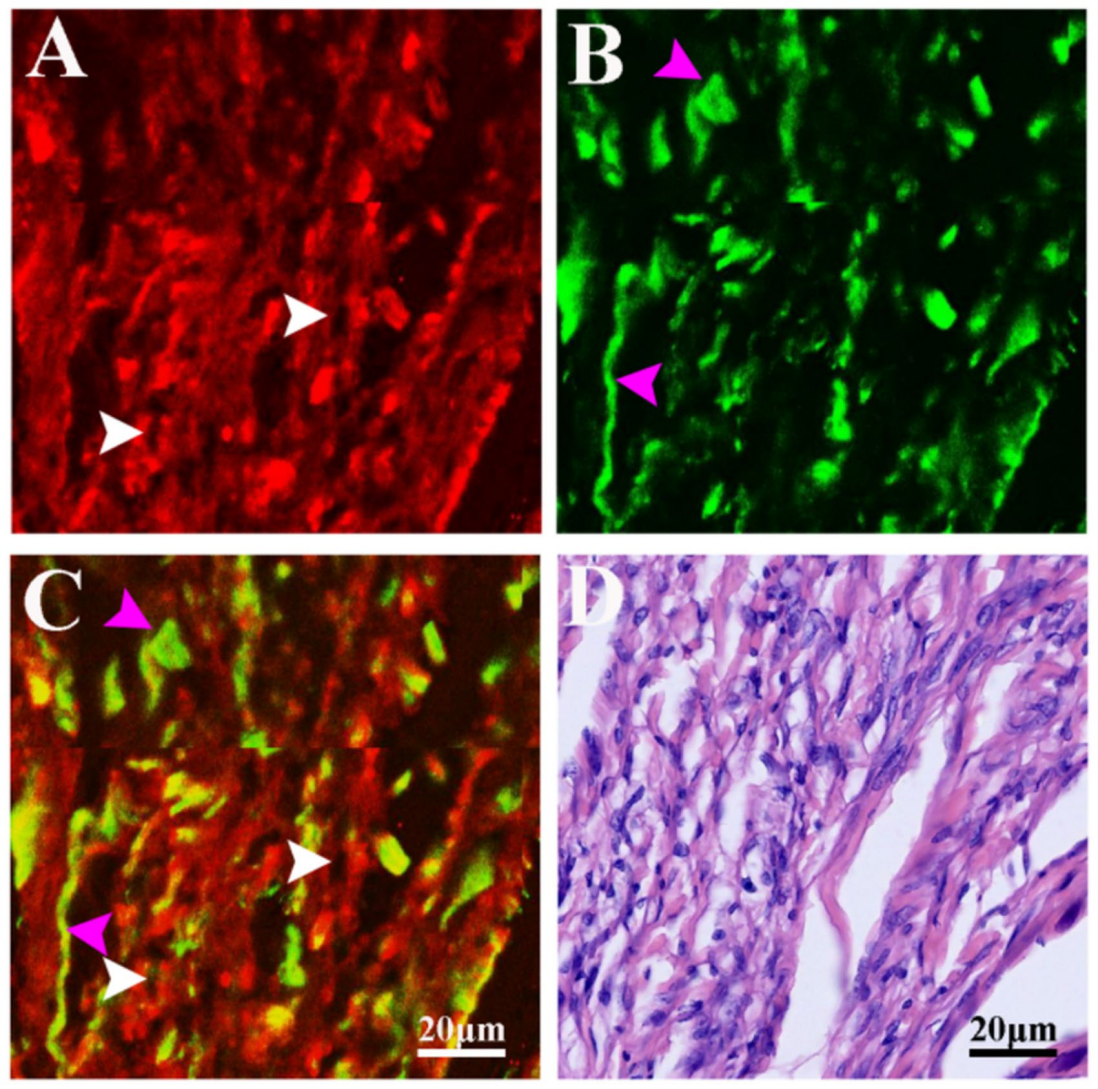
The magnified TPEF, SHG, TPEF/SHG overlaid and corresponding H&E images of the selected area (yellow line box) in Fig. S3C. **(A)** Magnified TPEF image; **(B)** magnified SHG image; **(C)** magnified TPEF/SHG overlaid images; **(D)** corresponding H&E stained images. White arrows: cells; purple arrows: collagen fiber.

To assess the capability of MPM in visualizing the microstructures of fibrous meningioma, we conducted multichannel-mode imaging on unstained fibrous meningioma tissues. Supplementary Figure 3 presents a typical TPEF image, SHG image, TPEF/SHG overlaid image of fibrous meningioma alongside its corresponding H&E stained image. As depicted in Supplementary Figure 3, the fibrous meningioma tissue is rich in collagen fibers. And, a large number of meningioma cells scatter in the collagen-rich matrix. More detailed structural information of fibrous meningioma can be observed at higher magnification, as shown in Figure 3 (a magnified view of the area indicated by the white box in Supplementary Figure 3C).

As illustrated in Supplementary Figures 3A–C, fibrous meningiomas are rich in collagen, with some collagen fibers aggregating into bundles structures. These collagen fibers generate SHG signals and also emit TPEF signals, appearing yellow (indicated by purple arrows) in the TPEF/SHG overlaid images (Supplementary Figure 3C).

Figures 3A–C provide detailed views of the cells within fibrous meningiomas. The nuclei, which lack fluorescent signals (indicated by white arrows), appear as dark spots. In contrast, the cell outlines are clearly delineated by the TPEF signals from NADH and FAD in the cytoplasm. The nuclei of these cells exhibit distinct heterogeneity, varying in size and shape. These morphological features of collagen and cells are consistent with the structural characteristics observed in the corresponding H&E stained images. However, collagen fibers in the stroma are not as clearly displayed in the H&E-stained images as they are in the SHG image and TPEF/SHG overlaid image.

### 3.3 Visualization of fibrous meningiomas using lambda-mode of MPM

We also performed spectroscopic imaging of meningioma samples using the lambda mode of MPM. Supplementary Figure 4 displays a representative 32-channel spectral image of fibrous meningiomas. The spectral images were captured across a wavelength range of 382–714 nm, with 32 channels and an interval of 11 nm. Among these channels, the 404 nm channel exhibited the strongest SHG signal, while the 511 nm channel showed the highest TPEF signal.

**Figure 4:**
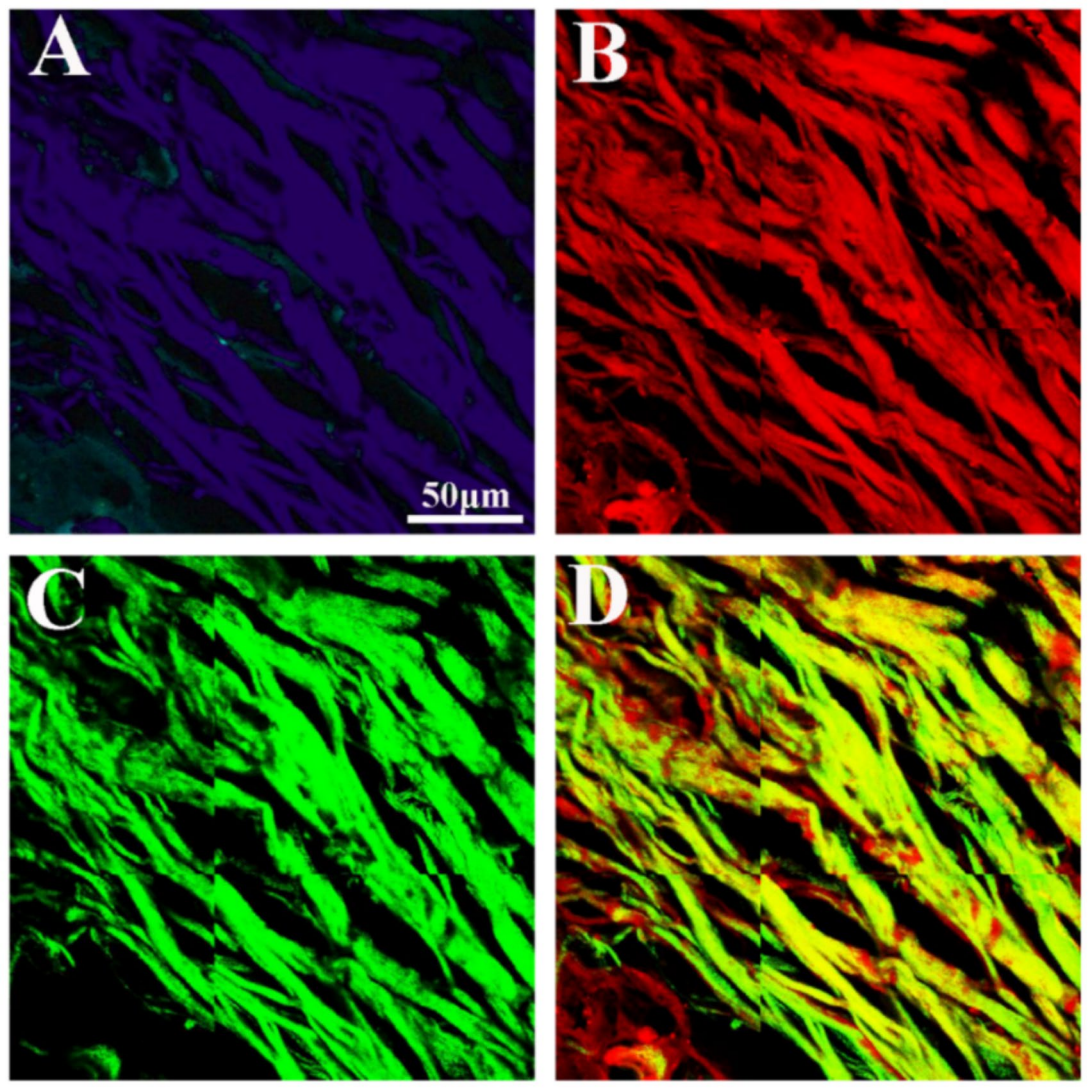
The 32 channels combined spectral image and corresponding TPEF image, SHG image, TPEF/SHG overlaid image of fibrous meningioma. **(A)** The 32 channels combined spectral image; **(B)** corresponding TPEF image; (B) corresponding SHG image; **(C)** corresponding TPEF/SHG overlaid images.

Figure 4 presents the combined spectral images and the corresponding overlaid TPEF/SHG images of fibrous meningiomas. These combined spectral images display two primary colors: purple and blue. The purple color indicates the SHG signal emitted by collagen fibers, whereas the blue color corresponds to the TPEF signal. Consequently, these high-contrast and high-resolution images derived from the combined spectral imaging provide detailed information for evaluating the morphological characteristics of fibrous meningiomas.

### 3.4 Quantification analysis of fibrous meningiomas

**Figure 5:**
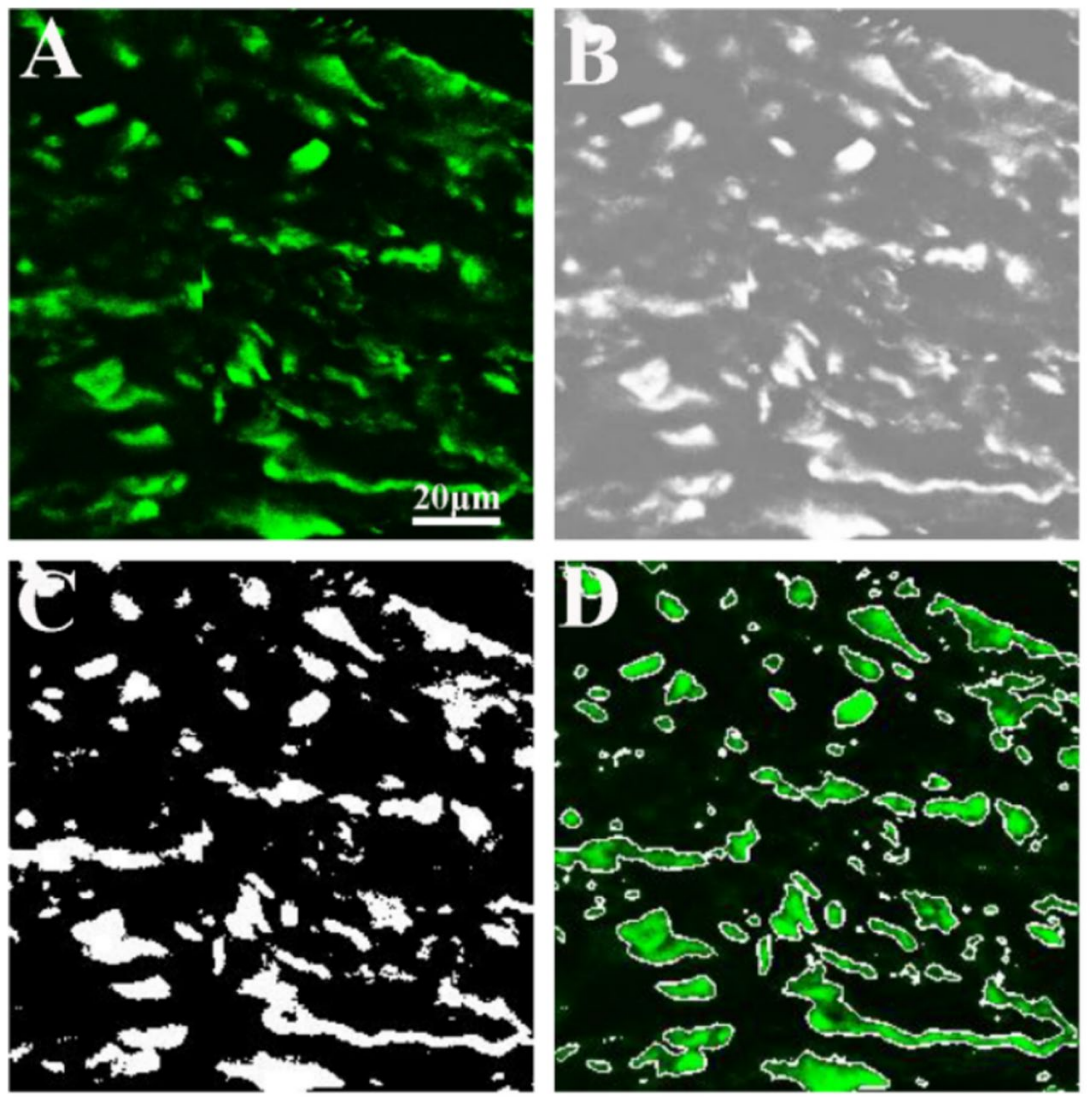
Image analysis of collagen fiber in fibrous meningioma using 404 nm channel spectral image. **(A)** Original 404 nm channel spectral image; **(B)** image after image enhancement processing; **(C)** image after morphological processing; **(D)** final image analysis results.

**Figure 6:**
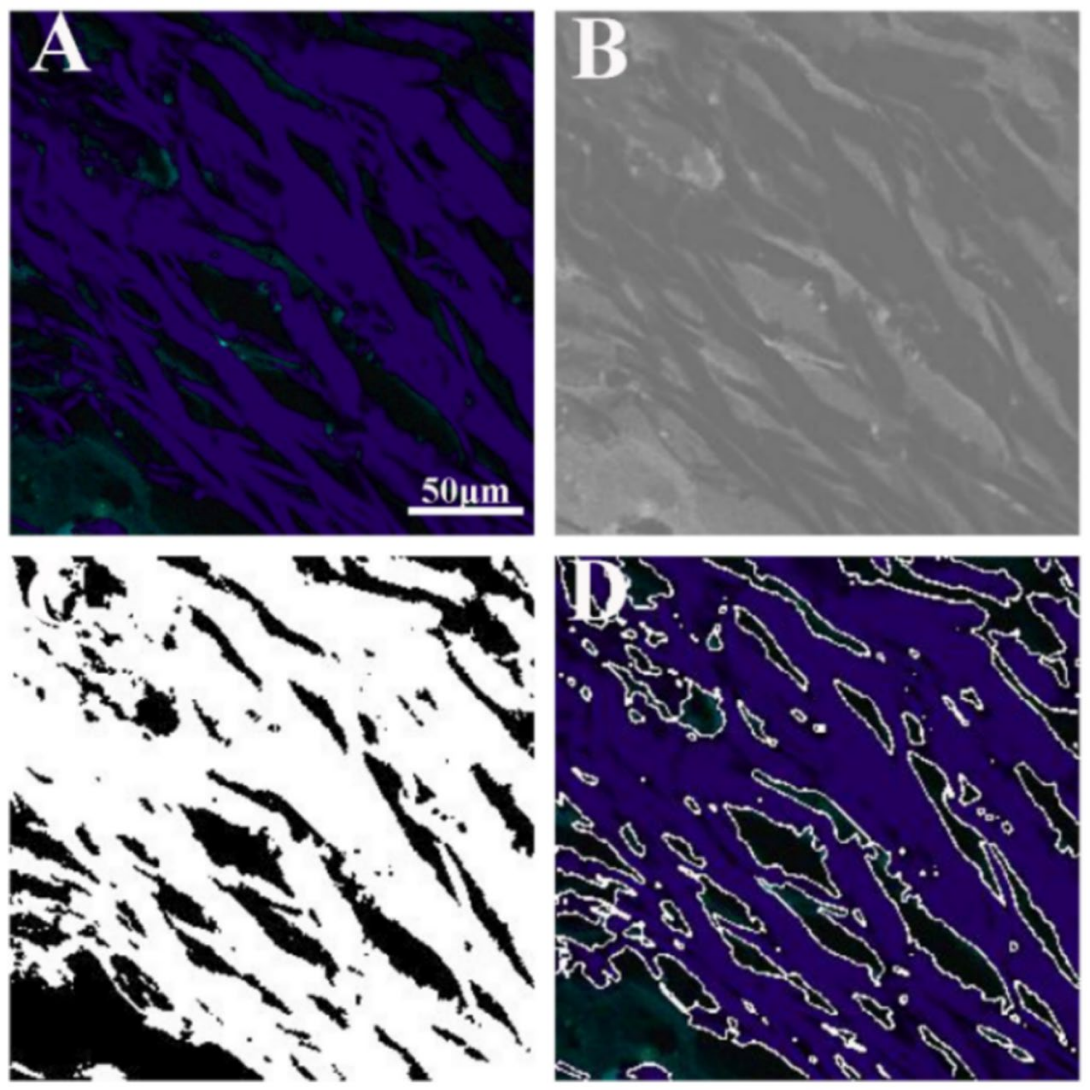
Image analysis of collagen fiber in fibrous meningioma using the 32 channels combined spectral image. **(A)** Original the 32 channels combined spectral image; **(B)** image after image enhancement processing; **(C)** image after morphological processing; **(D)** final image analysis results.

Figure 5 illustrates the results of image analysis of collagen fibers in fibrous meningioma using SHG imaging, while Figure 6 presents the results using the combined 32-channel spectral image. Figures 5, 6 show the original SHG image (Figure 5A), the combined 32-channel spectral image (Figure 6A), the enhanced images (Figures 5B, 6B), the images after morphological processing (Figures 5C, 6C), and the final segmentation results (Figures 5D, 6D). At the cellular level, the final segmentation results closely match the original images. Quantitative results revealed that the value using SHG imaging was 0.537 ± 0.131, while using the combined 32-channel spectral image, the values were 0.503 ± 0.133. These results demonstrate that MPM combined with image analysis can automatically locate collagen fibers and quantify collagen content in fibrous meningiomas, providing valuable quantitative data.

## 4 Discussion

Currently, intraoperative diagnosis of fibrous meningioma still depends on frozen-section H&E histology. This workflow is time-consuming because each frozen section must be cut, frozen, stained and examined, and it sometimes introduces freezing artifacts, tissue curling and cellular distortion that can compromise diagnostic certainty. MPM, based on nonlinear femtosecond-laser technology, is expected to eliminate these drawbacks by offering real-time, label-free histological images of multiple regions of interest within seconds, thereby improving both surgical efficiency and diagnostic reliability compared with frozen-section H&E staining.

In this study, our results demonstrated that MPM has the potential to visualize the microstructure of fibrous meningiomas through TPEF signals from cells and SHG signals from collagen. Importantly, this capability is further enhanced by MPM’s ability to exploit endogenous contrast: by simultaneously capturing SHG and TPEF emission, the system can produce composite images that immediately emphasize the architectural hallmarks of fibrous meningiomas, permitting accurate on-table identification. Although collagen distribution is likewise accessible with conventional trichrome staining, that approach demands tissue excision and a multi-day processing window, making it impractical in the surgical setting. MPM, in contrast, furnishes rapid, label-free collagen visualization that fits naturally into the operative workflow. The synergy of high-speed acquisition and multichannel detection therefore underscores MPM’s unique suitability for real-time, objective appraisal of fibrous-meningioma collagen distribution.

By using the multichannel mode of MPM, high-contrast and high-resolution images obtained from fibrous meningioma tissues can provide detailed information to assess the morphological characteristics of cells and collagen. We also performed spectroscopic imaging of fibrous meningioma samples using the lambda mode of MPM, and the results showed that, similar to TPEF/SHG overlaid images, combined spectral images can also effectively depict the morphological details of fibrous meningiomas. Coupled with automated image-analysis algorithms, MPM rapidly identifies collagen fibres and quantifies their content, offering neuropathologists and neurosurgeons a fast, objective measure of collagen deposition in fibrous meningiomas.

Although MPM already permits accurate identification of fibrous meningioma, its ultimate clinical value lies in cure. Current options—radio-frequency ablation and endoscopic resection—each have distinct drawbacks: ablation provides no tissue for histological verification, whereas conventional endoscopy frequently causes parenchymal deformation, traction injury, and electrical interference with intra-operative monitoring. An ideal system would integrate real-time diagnosis with precise, atraumatic excision. Femtosecond-pulse lasers fulfil this requirement: when operated at amplified energies they produce plasma-mediated, non-thermal photo-disruption that cleanly severs cells or sub-cellular structures without collateral coagulation or char (König, 1999; Hild et al., 2008). Coupling such a laser to the same delivery fibre as the imaging source would allow MPM to visualize tumor margins at low power and immediately ablate residual fibrous meningioma at high power, while preserving adjacent vessels and cranial nerves. The present study establishes the diagnostic component of this platform; our next step is to optimize multiphoton ablation parameters for safe, image-guided removal of fibrous meningioma.

## 5 Conclusion

In summary, this study highlights the potential of MPM for visualizing fibrous meningiomas using multichannel and lambda modes. MPM provides high-resolution, label-free images that reveal detailed collagen and cellular structures. Multichannel mode captures SHG and TPEF signals, while lambda mode offers comprehensive spectral analysis for identifying intrinsic components. Image analysis quantifies collagen content, aiding surgical planning and prognosis. Compared to traditional methods, MPM offers real-time, high-contrast imaging with superior resolution, overcoming limitations like tissue damage and labor-intensive processing. It is expected that MPM can be used in clinical diagnosis of fibrous meningiomas in the future with the development of a new MPM endoscope system.

## Data Availability

The datasets presented in this article are not readily available because the raw and processed multiphoton-microscopy datasets generated in this study are not publicly available owing to patient-privacy regulations. Access to any de-identified data will be granted only after written permission from the corresponding author and execution of a formal data-use agreement. Requests to access the datasets should be directed to NF, fangna2005684@163.com.
