# Supplementary material for: Multiphoton microscopy imaging of fibrous meningiomas based on the combination of multichannel mode and lambda mode
Source: Front Neurosci. 2025 Oct 31;19:1680408. doi: 10.3389/fnins.2025.1680408 (PMC12615439; doi:10.3389/fnins.2025.1680408)
Supplement: Supplementary file 1 [file Supplementary_file_1.docx]

Supplementary Material

# Supplementary Data

## Supplementary Figures

##
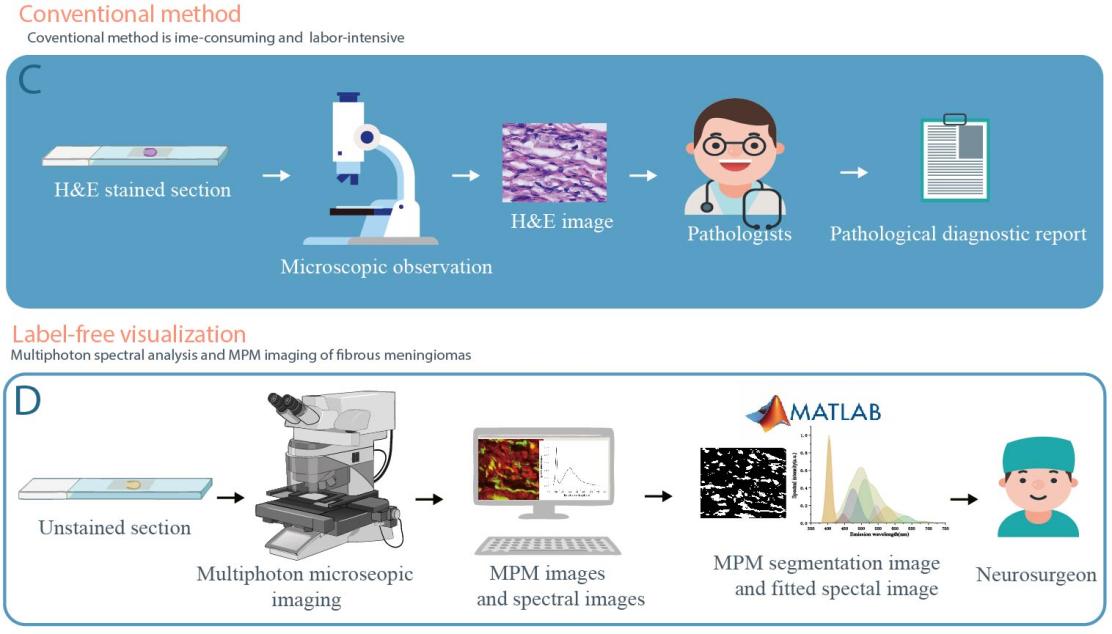


## Supplementary Figure 1. Multiphoton microscopy for visualization of fibrous meningioma. (A) Conventional method for visualizing fibrous meningioma. (B) Multiphoton microscopy for visualizing fibrous meningioma.


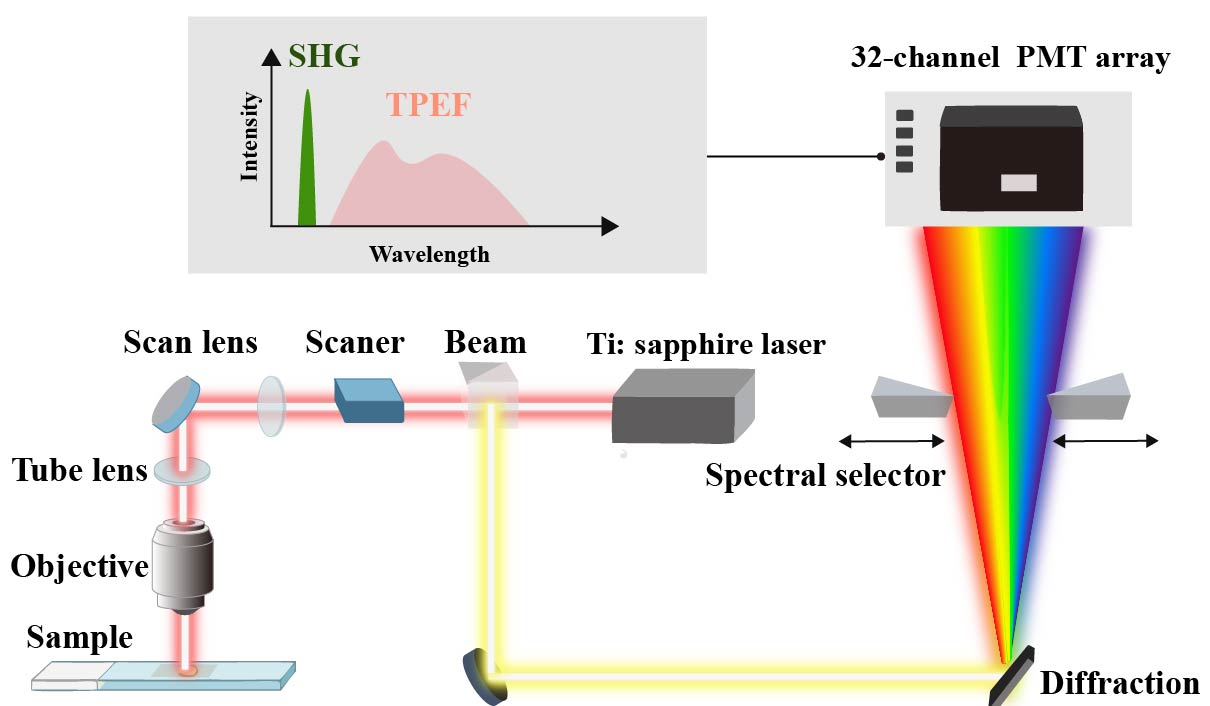


**Supplementary Figure 2.** Sketch of multiphoton microscopy system.


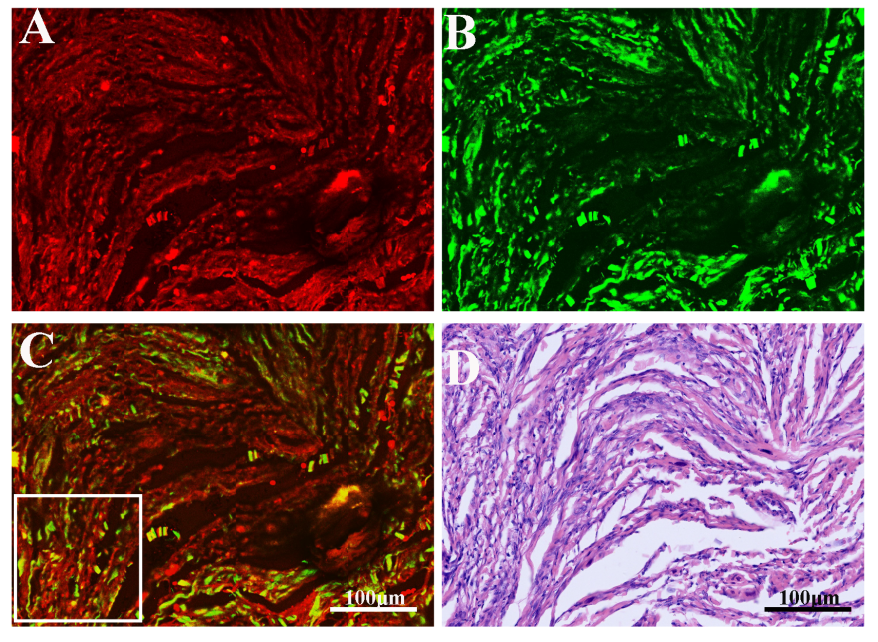


**Supplementary Figure 3.** Typical TPEF image, SHG image, TPEF/SHG overlaid image and the corresponding H&E stained images of fibrous meningioma. (A) TPEF image; (B) SHG image; (C) TPEF/SHG overlaid images; (D) corresponding H&E stained image.


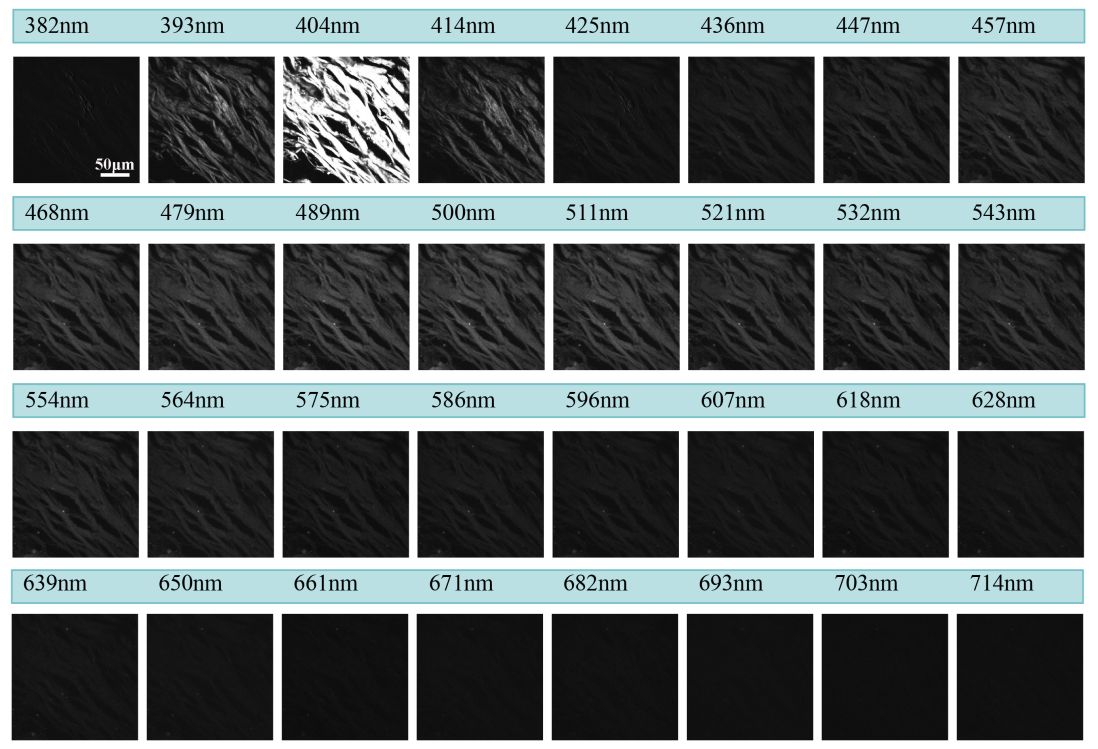


**Supplementary Figure 4.** The 32-channels spectral image of fibrous meningiomas.
